# EPHA2 Promotes the Invasion and Migration of Human Tongue Squamous Cell Carcinoma Cal-27 Cells by Enhancing AKT/mTOR Signaling Pathway

**DOI:** 10.1155/2021/4219690

**Published:** 2021-03-24

**Authors:** Fengqin Wang, Hanzhong Zhang, Zhigang Cheng

**Affiliations:** Department of Stomatology, The Central Hospital of Wuhan, Tongji Medical College, Huazhong University of Science and Technology, 26 Shengli Street, Wuhan 430014, China

## Abstract

EPHA2 is a member of the ephrin receptor tyrosine kinase family and is closely related to the malignant tumor progression. The effect of EPHA2 on OSCC is not clear. This study explored the role of EPHA2 and AKT/mTOR signaling pathways in Cal-27 cell invasion and migration. The expression of EPHA2 and EPHA4 in human OSCC and normal oral tissue was detected by immunohistochemistry. EPHA2-overexpressing and EPHA2-knockdown Cal-27 cells were established, and the cells were treated with an AKT inhibitor (MK2206) and mTOR inhibitor (RAD001). The expression of EPHA2 was detected by qRT-PCR, cell proliferation was evaluated by MTT assay, cell migration and invasion were examined by scratch and Transwell assay, and cell morphology and apoptosis were assessed by Hoechst 33258 staining. Western blot was performed to detect the expression of proteins related to AKT/mTOR signaling, cell cycle, and pseudopod invasion. EPHA2 and EPHA4 were highly expressed in clinical human OSCC. Overexpression of EPHA2 promoted the proliferation, migration, and invasion of Cal-27 cells, inhibited cell cycle blockage and apoptosis, and enhanced the activity of the AKT/mTOR signaling pathway. MK2206 (AKT inhibitor) and RAD001 (mTOR inhibitor) reversed the effect of EPHA2 overexpression on the biological behavior of Cal-27 cells. EPHA2 promotes the invasion and migration of Cal-27 human OSCC cells by enhancing the AKT/mTOR signaling pathway.

## 1. Introduction

Oral squamous cell carcinoma (OSCC) is the most common malignancy in the oral and maxillofacial region. In China, the incidence of OSCC is between 36/100,000 and 8.0/100,000, and the five-year survival rate is 50% [1]. OSCC has a high local recurrence rate, with the tendency to develop early lymph node metastasis and late metastasis to the lung, liver, bone, and other organs [2]. As tumor metastasis is the main cause of cancer death, the discovery of key regulatory molecules in the process of cancer metastasis and identification of effective diagnostic markers or therapeutic targets have become highlighted topics of research.

EPHA2 is a member of the EPH receptor tyrosine kinase family. It is highly expressed in glioblastoma, ovarian cancer, and cholangiocarcinoma and is closely related to malignant tumor progression [3]. Recent studies have shown that EPHA2 promotes epithelial-mesenchymal transition and cell migration by enhancing the Wnt/*β*-catenin signaling pathway. Likewise, EPHA2 upregulates the expression of CyclinD1 and promotes cell cycle progression, thereby enhancing the proliferation of gastric cancer cells [4]. Furthermore, EPHA2 is significantly and frequently mutated in OSCC [5] and associated with the MAPK/ERK, JAK/STAT, and mTOR/AKT signaling pathways [6]. Everolimus, also known as RAD001, is a mammalian target for specific inhibitors of rapamycin (mTORC1) [7]. MK2206 is an allosteric inhibitor of AKT, which can decrease p- (Ser473-) AKT and p- (Thr308-) AKT in a dose-dependent manner [8].

EPHA2 has been shown to activate the protein kinase B-mammalian rapamycin target protein (AKT/mTOR) signaling pathway [9], but whether EPHA2 is related to AKT/mTOR signaling in OSCC is not clear. This study explored the effect of AKT/mTOR signaling and EPHA2 on the progression of OSCC to provide a theoretical basis for prognostic evaluation and targeted therapy of OSCC.

## 2. Materials and Methods

### 2.1. Tissues and Cells

Human OSCC (10 cases) and normal oral tissue (5 cases) were collected from the Central Hospital of Wuhan. Human tongue squamous carcinoma Cal-27 cells were purchased from the American Type Culture Collection (Rockefeller, Maryland, USA). A frozen tube containing 1 ml of cell suspension was rapidly shaken and thawed in a water bath at 37°C and mixed with 4 ml of Dulbecco's modified Eagle medium containing 10% fetal bovine serum (Gibco, Shanghai, China). The cells were centrifuged for 4 min at 1000 rpm, the supernatant was discarded, and 2 ml of medium was added. Then, the cells were cultured overnight, and the medium was changed on the next day, after which the cell density was measured. The experiment was approved by the Ethics Committee of Wuhan Central Hospital and fully respected the right of informed consent of the subjects and their families.

### 2.2. Protocol

The expression of EPHA2 and EPHA4 was analyzed in OSCC and normal oral tissues. To explore the effect of EPHA2 on the biological behavior of OSCC cells, the cells were subjected to overexpression of inhibition of EPHA2. To verify whether the AKT/mTOR signaling pathway participated in the mechanism underlying the action of EPHA2, the AKT inhibitor MK2206 (0.1 *μ*mol/L, S1078, Selleck, TX, USA) and mTOR inhibitor RAD001 (20 nmol/L, 159351-69-6, Aladdin, Shanghai, China) were added.

### 2.3. Construction of Overexpression and Inhibition Plasmids

The overexpression EPHA2 plasmid was constructed using pcDNA3.1 containing Amp resistance (Addgene, LGC Standards Teddington, UK). The 2931-bp EPHA2 target gene was amplified using forward (5′-CTAGCTAGCTAGATGGAGCTCCAGGCA-3′) and reverse (5′-CCGCTCGAGCGGTCAGATGGGGATCCCC-3′) primers. NheI and EcoRI enzymes were used to slice and recover the plasmids. The EPHA2 target gene was connected to the slicing plasmid, transformed, and sequenced. The EPHA2 inhibition plasmid was constructed using pSIREN containing Amp resistance (Addgene, UK). The sense was 5′-GATCCGCGTCATCTCCAAATACAAGCTTCAAGAGAGCTTGTATTTGGAGATGACGCTTTTTG-3′, and the antisense was 5′-AATTCAAAAAGCGTCATCTCCAAATACAAGCTCTCTTGAAGCTTGTATTTGGAGATGACGC-3′. Bam HI and EcoRI enzymes were used to slice and recover the plasmid. The target gene was connected, transformed, and sequenced. The overexpression and inhibition plasmids were introduced into Cal-27 cells by transient transfection using Lipofectamine™ 2000. The overexpression groups were oeEPHA2 and oeNC, and the inhibition groups were shEPHA2 and shNC, respectively.

### 2.4. Immunohistochemistry

Tissues at 0.2–0.3 cm were fixed in 10% formalin for 48 h. The tissues were placed in an embedding box and dehydrated for 10 h with an automatic tissue dehydrator. Then, the tissues were embedded in paraffin, placed in a freezer for 1 h, and sectioned at 5 *μ*m. The sections were heated at 65°C for 1 h and immersed twice in xylene for 15 min each. Dehydration at a concentration gradient from 100% to 75% alcohol was carried out, and 0.01 M sodium citrate buffer was used for repair for 15 min in 125°C at 10^3^ kPa. After room temperature cooling, the sections were washed three times with phosphate-buffered saline (PBS). The sections were then incubated in 3% H_2_O_2_ in a humidified box for 10 min, blocked with 0.5% bovine serum albumin for 30 min, incubated with primary antibodies overnight at 4°C, and incubated with MaxVision II for 30 min at room temperature. Diaminobenzidine (Bioswamp, Wuhan, China) was added to stain the sections, and when a color change was observed, the dye solution was washed and the sections were counterstained with hematoxylin for 3 min. Image acquisition was carried out using Leica Application Suite (MD1000, Frankfurt, Germany). The antibodies used were rabbits anti-EPHA2 (1 : 50, PAB35826), rabbit anti-EPHA4 (1 : 50, PAB35096), and MaxVision™ HRP-Polymer anti-Mouse/Rabbit IHC kit (1 : 200, PAB160022), all purchased from Bioswamp (Wuhan, China).

### 2.5. 3-(4,5-Dimethyl-2-Thiazolyl)-2,5-Diphenyl-2-H-Tetrazolium Bromide (MTT) Assay

Cells were seeded in 96-well plates at 2 × 10^3^ cells per well. 20 *μ*L of MTT (5 mg/mL) solution (Bioswamp, Wuhan, China) was added to each well, and the cells were incubated for 0, 12, 24, 48, or 72 h. Then, the medium was removed, and 150 *μ*L of dimethyl sulfoxide (Sigma, CA, USA) was added into each well. After fully mixing, the optical density was measured at 490 nm using a plate reader (Multiskan FC, Thermo, Massachusetts, USA).

### 2.6. Wound Healing Assay

Cells were seeded in 6-well plates at 5 × 10^5^ cells per well. Before cell seeding, a line was drawn at the bottom of the well plate every 0.5 cm using a marker, and four lines were drawn in each well. On the next day, a scratch was made perpendicular to the well using a pipette tip against a ruler. The cells were washed with PBS, and culture medium was added to the wells. Photographs were taken at 0, 24, and 48 h to observe scratch healing. The Image J software was used to measure the scratch area and calculate the cell migration ability. Scratch healing rate = (scratch area at 0 h–scratch area at 24 h)/scratch area at 0 h × 100%.

### 2.7. Transwell Assay

Cal-27 cells (1 × 10^4^) were seeded with serum-free medium on the top chamber of a Transwell insert, and medium containing serum was added to the bottom chamber. After 48 hours of incubation at 37°C in 5% CO_2_, the cells were fixed using 4% formaldehyde and stained with 0.1% crystal violet. The number of cells that have passed through the chamber was observed under a microscope and photographed, and cell migration ability was calculated. For invasion, a Matrigel filter was placed in the Transwell chamber. Invasive ability was evaluated by counting the number of cells that passed through the Matrigel filter.

### 2.8. Hoechst 33258 Assay

Monolayer cells (about 50-80%) were immobilized with 4% paraformaldehyde for 10 min and washed twice with PBS in 12-well plates. 0.5 ml of Hoechst 33258 dye solution was added, and the cells were stained at room temperature for 5 min. After five washes with PBS, cell morphology and apoptosis were observed by fluorescence microscopy (DMIL LED, Leica, Germany).

### 2.9. Flow Cytometry

Cells in the logarithmic growth phase were incubated in 6-well plates at 2 × 10^5^ cells/ml. After 48 h of incubation, the cell density was adjusted to 4 × 10^5^ cells/ml. After two washes with PBS, the cells were suspended in 500 mL of PBS, and 5 *μ*L of Annexin-FITC and 5 *μ*L of propidium iodide (PI) (BD, NJ, USA) were added to each well. After fully mixing, the cells were incubated in the absence of light for 30 min at room temperature. Apoptotic cells were immediately detected by flow cytometry. For cell cycle detection, 400 *μ*L of PI (50 *μ*g/mL) was added in the wells and flow cytometry was performed after mixing at room temperature for 10 min.

### 2.10. Western Blot

Cells were washed twice with precooled PBS and lysed at 4°C using radioimmunoprecipitation assay buffer containing protease and phosphatase inhibitors (Beyotime, Shanghai, China). The cells were then heated at 95°C for 10 min and centrifuged at 12000 rpm for 10 min, and protein content was quantified. Lower and upper electrophoresis gels were prepared at 12% and 5%, respectively, and the protein solution was denatured in boiling water for 10 min. 20 *μ*g of proteins was subjected to sodium dodecyl sulfate-polyacrylamide gel electrophoresis and transferred onto polyvinylidene fluoride membranes. The membranes were blocked with 5% skimmed milk powder at room temperature for 2 h and incubated with primary antibodies overnight at 4°C. After three washes with PBS-Tween 20 (PBST), secondary antibodies labeled with horseradish peroxidase (HRP) were added and the membranes were incubated at room temperature for 1 h, followed by three washes with PBST. Enhanced chemiluminescent reagent (Millipore, Massachusetts, USA) was added, and protein bands were detected in a fully automatic chemiluminescent analyzer. Antibody information is listed in Table 1.

### 2.11. Reverse Transcriptase-Polymerase Chain Reaction (qRT-PCR)

Total RNA was extracted by Trizol. After precipitation with chloroform, isopropanol, and 75% ethanol, total RNA was dissolved in 40 *μ*L of DEPC water. The RNA was reverse-transcribed into cDNA using a reverse transcription kit (TaKaRa, Dalian, China), and the prepared cDNA was amplified using the SYBR Green PCR kit (KM4101, KAPA Biosystems, USA). The amplification system was as follows: 10 *μ*L of SYBR Green Mix, 5 mol of forward primer, 5 mol of reverse primer, 1 *μ*L of cDNA, and 8 *μ*L of double-distilled water for a total of 20 *μ*L. Reaction procedure is as follows: 95°C for 3 min; 39 cycles of 95°C for 5 s, 56°C for 10 s, 72°C for 25 s; 65°C for 5 s; 95°C for 50 s. The primers were EPHA2-forward (5′-CTGCTCGCCTGGATT-3′), EPHA2-reverse (5′-ACGGCTGTGAGGTAGTG-3′), GAPDH-forward (5′-CCACTCCTCCACCTTTG-3′), and GAPDH-reverse (5′-CACCACCCTGTTGCTGT-3′).

### 2.12. Statistical Analysis

All data were analyzed using the SPSS19.0 statistical software (IBM, NY, USA) and expressed as the mean ± standard deviation. Comparisons between groups were performed by one-way analysis of variance. *P* < 0.05 indicates statistical significance.

## 3. Results

### 3.1. Abnormally High Expression of EPHA2 and EPHA4 in Human Oral Squamous Cell Carcinoma

**Figure 1:**
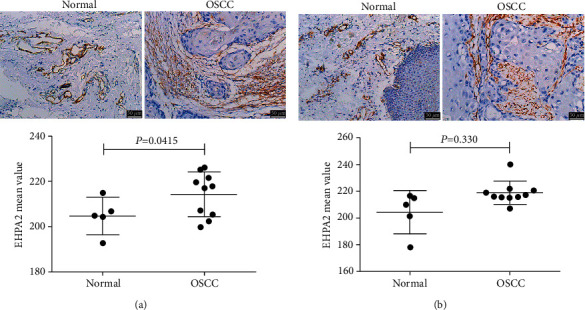
EPHA2 and EPHA4 were highly expressed in human OSCC tissue. (a) EPHA2 and (b) EPHA4 expression were detected by immunohistochemistry (scale bar = 50 *μ*m) and analyzed by SPSS19.0. *n* = 5 for normal oral tissue and *n* = 10 for human OSCC tissue. *P* < 0.05 indicates significant difference.

The expression of EPHA2 and EPHA4 in 10 cases of human OSCC was abnormally high compared with that in the 5 cases of normal oral cell tissues, as revealed by immunohistochemistry (Figure 1), and the difference was statistically significant (*P* < 0.05).

### 3.2. EPHA2 Overexpression and Knockdown Plasmids Were Successfully Constructed and Transfected into Cal-27 Cells

**Figure 2:**
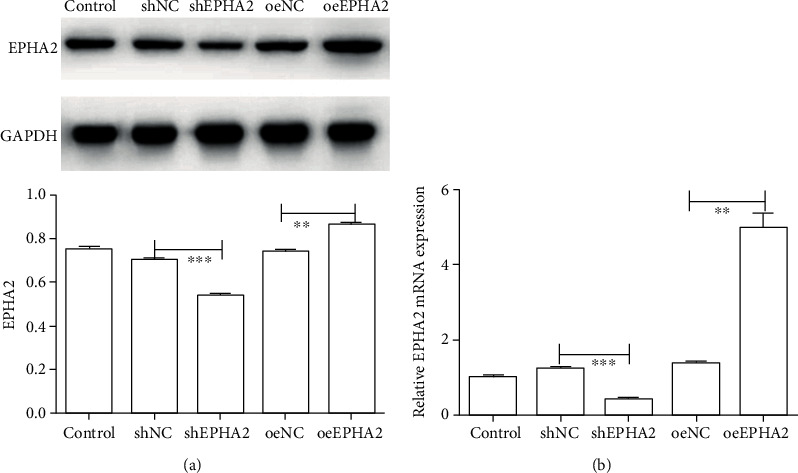
mRNA and protein expression of EPHA2 were analyzed in Cal-27 cells subjected to overexpression and knockdown of EPHA2. (a) Western blot detection of protein expression of EPHA2. (b) qRT-PCR analysis of mRNA expression of EPHA2. Control, Cal-27 cells; shNC, Cal-27 transfected with empty pSIREN vectors; shEPHA2, Cal-27 transfected with pSIREN-shEPHA2; oeNC, Cal-27 transfected with empty pcDNA3.1 vectors; oeEPHA2, Cal-27 transfected with pcDNA3.1-EPHA2. *n* = 3. ^∗∗^*P* < 0.01 and ^∗∗∗^*P* < 0.001 indicate significant difference.

The expression of EPHA2 was detected by qRT-PCR and Western blot (Figure 2). The results showed that the expression of EPHA2 was significantly increased in the oeEPHA2 group (*P* < 0.01) and significantly decreased in the shEPHA2 group (*P* < 0.001), suggesting that EPHA2 overexpression and knockdown were successful in Cal-27 cells.

### 3.3. Effect of EPHA2 Overexpression on Cal-27 Cell Proliferation Was Reversed by AKT/mTOR Inhibitors

**Figure 3:**
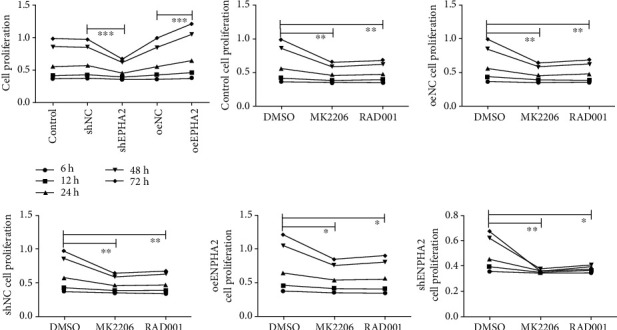
Overexpression of EPHA2 and AKT/mTOR inhibitor affected the proliferation of Cal-27 cells. MTT detection of the proliferation of Cal-27 cells at 6, 12, 24, 48, and 72 h. MK2206 is an AKT inhibitor, and RAD001 is an mTOR inhibitor. *n* = 3. ^∗^*P* < 0.05, ^∗∗^*P* < 0.01, and ^∗∗∗^*P* < 0.001 indicate significant difference.

The effect of EPHA2 on the proliferation of Cal-27 cells was examined using MTT assay (Figure 3). EPHA2 overexpression significantly promoted the proliferation of Cal-27 cells at 24, 48, and 72 h (all *P* < 0.001), while EPHA2 knockdown significantly inhibited the proliferation of Cal-27 cells at 24, 48, and 72 h (all *P* < 0.001). After MK2206 and RAD001 treatment, the effect of EPHA2 overexpression on the proliferation of Cal-27 cells was reversed.

### 3.4. Effect of EPHA2 Overexpression on Migration and Invasion of Cal-27 Cells Was Reversed by AKT/mTOR Inhibitors

**Figure 4:**
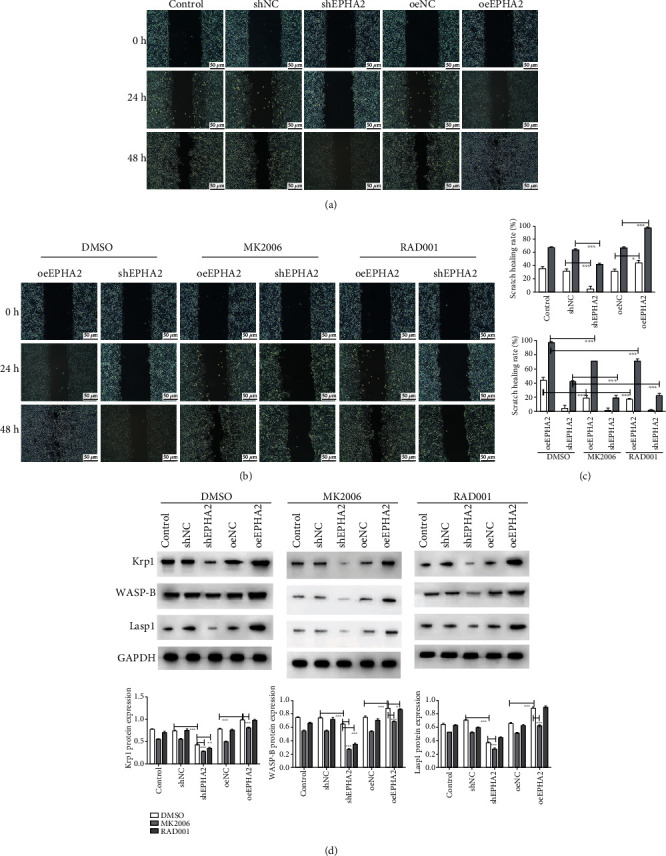
Detection of Cal-27 cell migration and invasive pseudopod-related proteins. (a) The migration of Cal-27 cells was assessed at 0, 24, and 48 h by scratch assay (scale bar = 50 *μ*m). (b) Cell migration was detected after MK2206 and RAD001 treatment (scale bar = 50 *μ*m). (c) Statistical analysis of scratch healing rate (%). *n* = 3. ^∗^*P* < 0.05 and ^∗∗∗^*P* < 0.001 indicate significant difference. (d) Western blot detection of invasive pseudopod-related proteins Krp1, WASP-B, and Lasp1. *n* = 3. ^∗^*P* < 0.05, ^∗∗^*P* < 0.01, and ^∗∗∗^*P* < 0.001 indicate significant difference.

**Figure 5:**
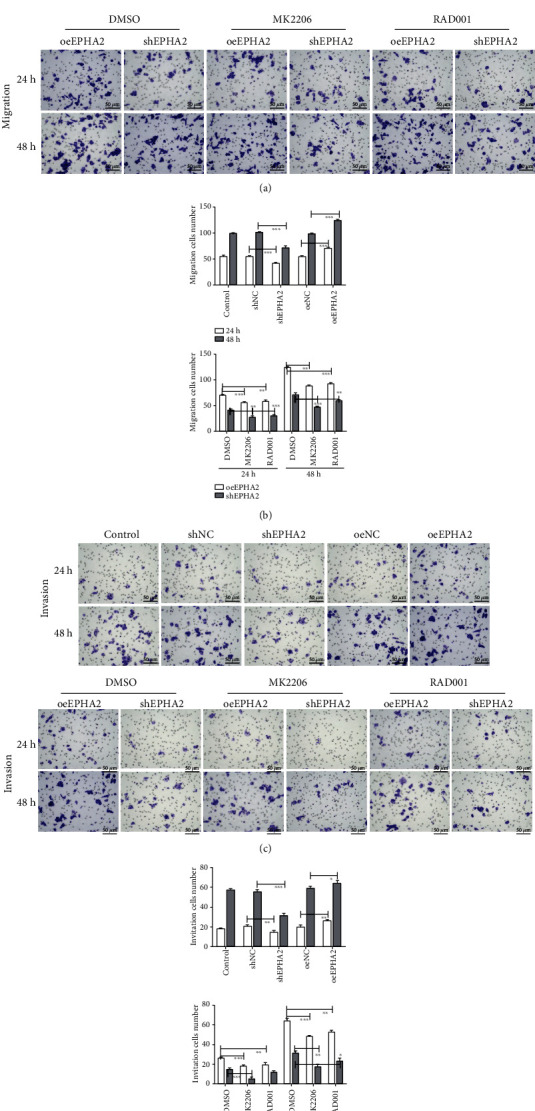
Transwell assay of Cal-27 cell migration and invasion. (a) The migration of Cal-27 cells was detected at 24 and 48 h (scale bar = 50 *μ*m), with or without MK2206 and RAD001 treatment. (b) The number of migrating cells was statistical analyzed. (c) The invasion of Cal-27 cells was detected at 24 and 48 h (scale bar = 50 *μ*m), with or without MK2206 and RAD001 treatment. (d) The number of invading cells was statistical analyzed. *n* = 3. ^∗^*P* < 0.05, ^∗∗^*P* < 0.01, and ^∗∗∗^*P* < 0.001 indicate significant difference.

The migration of Cal-27 cells was detected by scratch assay at 0, 24, and 48 h. EPHA2 overexpression significantly promoted cell migration (Figure 4(a)) at 24 and 48 h (*P* < 0.05 and *P* < 0.001), but MK2206 and RAD001 reversed the effect of EPHA2 and inhibited Cal-27 cell migration (Figure 4(b)) at 48 h (*P* < 0.001), but not RAD001 treatment at 24 h. Similarly, the migration of Cal-27 cells (Figure 5(a)) was detected by Transwell assay at 24 and 48 h, and the results were consistent with those of the scratch assay. In addition, Transwell assay (Figure 5(c)) showed that EPHA2 overexpression and knockdown significantly promoted and inhibited the invasion of Cal-27 cells, respectively, and the effect of EPHA2 was counteracted by MK2206 and RAD001. Overexpression of EPHA2 also significantly enhanced the activity of invasive pseudopod-related proteins Krp1, WASP-B, and Lasp1 (*P* < 0.001), while MK2206 had the opposite effect but not RAD001 (Figure 4(c)) on Krp1 and Lasp1 in the oeEPHA2 group.

### 3.5. EPHA2 Knockdown Promoted Cell Cycle Blockage and Apoptosis

**Figure 6:**
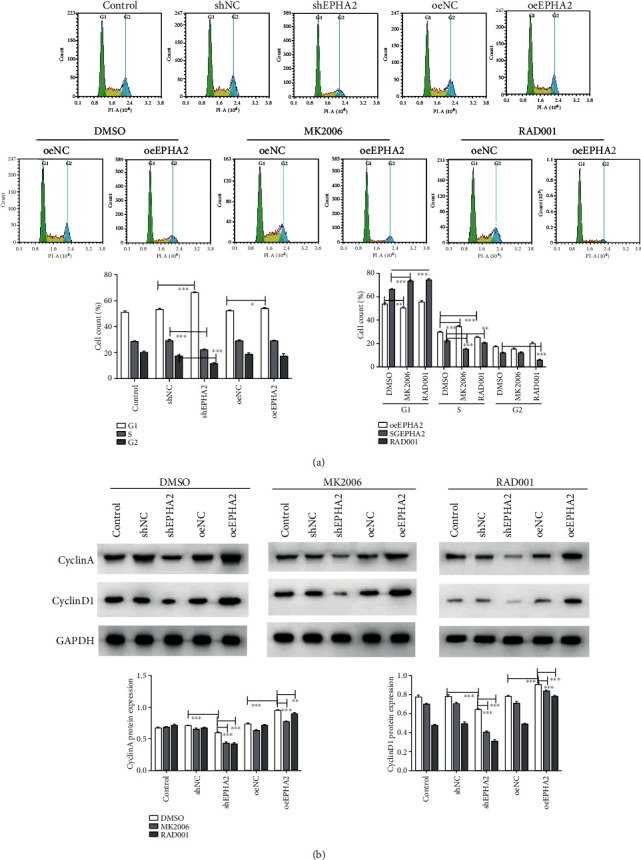
EPHA2 knockdown promoted cell cycle blockage in Cal-27 cells. (a) Cell cycle was examined by flow cytometry with or without MK2206 and RAD001 treatment. (b) Western blot detection of cell cycle-related proteins CyclinA and CyclinD1. *n* = 3. ^∗^*P* < 0.05, ^∗∗^*P* < 0.01, and ^∗∗∗^*P* < 0.001 indicate significant difference.

**Figure 7:**
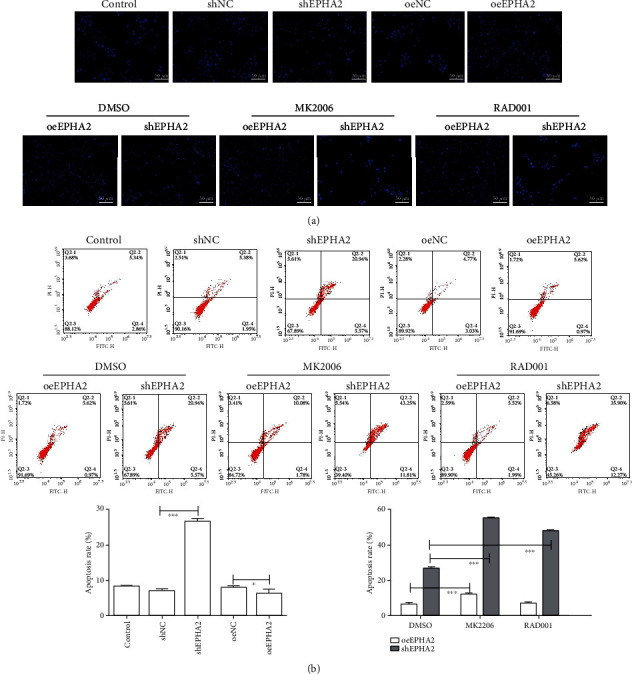
EPHA2 knockdown promoted the apoptosis of Cal-27 cells. (a) Evaluation of Cal-27 cell morphology and apoptosis by Hoechst 33258 staining (scale bar = 50 *μ*m). (b) Flow cytometry detection of Cal-27 cell apoptosis. *n* = 3. ^∗^*P* < 0.05 and ^∗∗∗^*P* < 0.001 indicate significant difference.

Flow cytometry was performed to detect changes in cell cycle after 48 h. EPHA2 knockdown significantly increased the proportion of cells in the G1 phase (*P* < 0.001) and decreased those in S and G2 (*P* < 0.001), but EPHA2 overexpression only increased the G1 phase (*P* < 0.05) (Figure 6(a)). After MK2206 and RAD001 treatment, the proportion of cells in the G1 phase was significantly increased (*P* < 0.01) by shEPHA2, whereas that in the S phase was markedly decreased (*P* < 0.01 and *P* < 0.001), indicating that cell cycle blockage by shEPHA2 was inhibited by MK2206 and RAD001. Western blot showed that EPHA2 knockdown downregulated the cell cycle-related proteins CyclinD1/A (Figure 6(b)). In addition, apoptotic cells were detected by flow cytometry after 24 hours. EPHA2 knockdown significantly promoted the apoptosis of Cal-27 cells (*P* < 0.001), while EPHA2 overexpression inhibits it (*P* < 0.05). MK2206 and RAD001 reversed the antiapoptotic effect of EPHA2 knockdown on Cal-27 cells, but RAD001 did not reverse the effect of EPHA2 overexpression on apoptosis (Figure 7(b)). Moreover, Hoechst 33258 staining showed that EPHA2 knockdown induced worse cell morphology than shNC, and EPHA2 overexpression showed the oppose trend. In addition, shEPHA2 induced cell apoptosis compared to shNC, while MK2206 and RAD001 treatment reversed the effect of EPHA2 overexpression on Cal-27 cell apoptosis and morphology.

### 3.6. EPHA2 Overexpression Enhanced the Activity of AKT/mTOR Signaling Pathway

**Figure 8:**
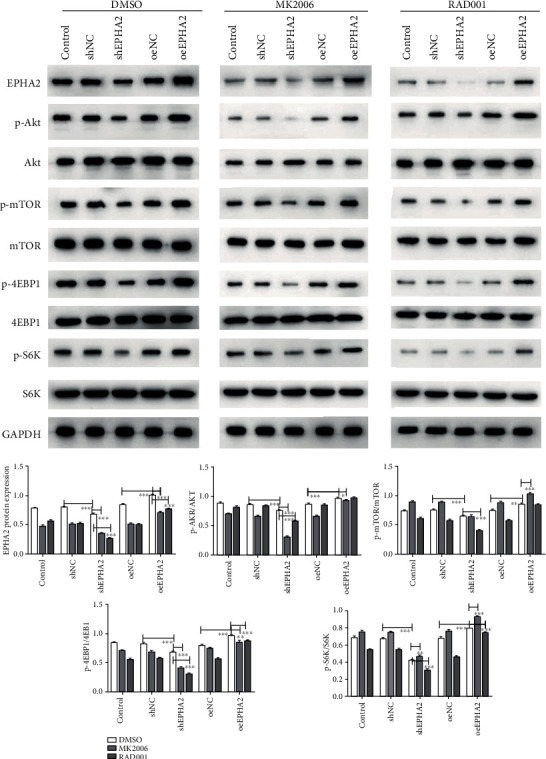
EPHA2 overexpression enhanced the activation of AKT/mTOR signaling. Western blot detection of the expression of EPHA2 and proteins related to AKT/mTOR signaling pathway (p-AKT, p-mTOR, p-4EBP1, and p-S6K). *n* = 3. ^∗^*P* < 0.05, ^∗∗^*P* < 0.01, and ^∗∗∗^*P* < 0.001 indicate significant difference.

EPHA2 overexpression significantly upregulated the activity of proteins in the AKT/mTOR signaling pathway, including p-AKT, p-mTOR, p-WASP-B, p-4EBP1, and p-S6K. EPHA2 knockdown had the opposite effect and inhibited the activity of AKT/mTOR signaling. In addition, MK2206 and RAD001 effectively reversed the effect of EPHA2, which verifies the regulatory role of EPHA2 on AKT/mTOR signaling (Figure 8).

## 4. Discussion

EPHA2, a member of the EPH family, plays an important role in tumorigenesis, angiogenesis, and intercellular adhesion [10]. Its expression is closely related to the degree of esophageal cancer differentiation and regional lymph node metastasis and is negatively related to clinical survival rate [11]. EPHA2 is highly expressed in breast [12], colorectal [13], esophageal [14], and prostate cancer [15]. In ovarian cancer, higher EPHA2 expression corresponds to more aggressive tumors. In turn, higher microvessel density in tumors signifies greater tumor malignancy [16]. In addition, overexpression of EPHA2 can regulate the proliferation, migration, invasion, and morphology of gastric cancer cells by upregulating TCF4, CyclinD1, and c-Myc in the Wnt/*β*-catenin signaling pathway [17]. It has been found that the positive expression of EPHA2 in OSCC was significantly higher than that in normal tissues, and EPHA2 expression at different clinical and pathological stages also showed significant differences, indicating that EPHA2 may be involved in the occurrence of OSCC [5]. EPHA2 binds to its ligand Ephrin A1 in normal tissues and acts on intracellular signal transduction molecules, regulates information transduction, and maintains cell-to-cell adhesion. However, EPHA2 cannot bind to its ligand efficiently in cancer tissues, resulting in abnormal EPHA2 signal transduction and cancer cell adhesion. It is easy to dissociate because of its low affinity and increased invasiveness, thus promoting the infiltration and metastasis of cancer tissues [18–20]. Our study first verified the abnormally high expression of EPHA2 and EPHA4 in OSCC, and the difference was statistically significant. By examining the proliferation, migration, invasion, and apoptosis of Cal-27 cells subjected to EPHA2 overexpression and knockdown, we found that EPHA2 overexpression inhibited G1 phase blockage by upregulating the expression of CyclinD1 and CyclinA. EPHA2 also promoted cell migration and invasion by upregulating the expression of invading pseudopod-related proteins (Krp1, WASP-B, and Lasp1). It has been proved that EPHA2 promotes the migration and invasion of human OSCC, but the mechanism underlying the effect of EPHA2 on the biological behavior of OSCC cells remains unclear.

The AKT/mTOR signaling pathway controls many important cellular biological processes in tumorigenesis and development, including maintenance of protein synthesis, cell survival, growth, metastasis, apoptosis, metabolism, angiogenesis, and cell cycle regulation. Studies have suggested that AKT/mTOR inhibition suppresses the proliferation, migration, and survival of cancer cells while strengthening the immune surveillance of tumors by preventing immunosuppressive pathways from activating and enhancing the innate immunity against tumors [21–23].

It has been reported that EPHA2 promotes malignant tumor progression by enhancing the AKT/mTOR signaling pathway. In cholangiocarcinoma, overexpression of EPHA2 enhanced AKT/mTOR signaling, thereby promoting the proliferation of CHO-CK cells in *vitro* and tumorigenicity in *vivo* [23]. In breast cancer, phosphorylated EPHA2 activated AKT through PI3K to participate in trastuzumab resistance [9]. In cervical cancer, EPHA2 activated AKT in a RhoG-dependent manner to promote the survival of HeLa cells [24]. In addition, AKT can reverse-regulate p-EPHA2 to promote malignant proliferation of cancer cells. Studies have shown that AKT promotes the migration and invasion of glioma and prostate cancer cells by enhancing the phosphorylation of serine 897 of EPHA2 [25]. In this study, Cal-27 cells were treated by MK2206 and RAD001. Only the AKT inhibitor reversed the effect of EPHA2 on G1 phase blockage, suggesting that EPHA2 promoted the proliferation and cell cycle of Cal-27 cells by enhancing AKT/mTOR signaling. CyclinD1 plays an important role in the regulation of cancer cell cycle [26], which is regulated by AKT/mTOR signaling. Our study found that inhibition of AKT/mTOR signaling antagonized the promotion of CyclinD1 and CyclinA expression in Cal-27 cells induced by EPHA2 overexpression, indicating that EPHA2 enhanced the expression of CyclinD1 and CyclinA in Cal-27 cells by enhancing AKT/mTOR signaling, thereby promoting cell cycle progression. Moreover, EPHA2 overexpression promoted the migration and invasion of Cal-27 cells by upregulating the expression of invasive pseudopod-related proteins. MK2206 reversed the expression of invasive pseudopod-related proteins, but RAD001 did not affect Krp1 and Lasp1 in the oeEPHA2 group. This may indicate that AKT but not mTOR activation was involved in the effect of EHPA2 on invasive pseudopod-related proteins. There may be other pathways involving mTOR activation affecting the invasion of Cal-27 cells.

Studies have shown that mTORC1 and mTORC2, two forms of mTOR, are regulated by different signal transduction pathways. mTORC1 is located downstream of AKT and directly regulates gene transcription through phosphorylation of ribosomal protein S6 kinase 1 (S6K1), 4EBP1, and its downstream target Mcl-1 [27], RNA splicing, and protein synthesis, while the downstream product S6K1 regulates AKT by negative feedback. mTORC2 is located upstream of AKT and regulates cell growth, survival, and migration by phosphorylating AKT and kinases in the AGC family [28]. At the same time, it regulates AKT via positive feedback [29]. In this study, we found that overexpression of EPHA2 upregulated p-AKT and p-mTOR and promoted the activation of downstream proteins p-WASP-B, p-S6K, and p-4EBP1, whereas EPHA2 knockdown had the opposite effect. The effect of EPHA2 was inhibited by MK2206 and RAD001.

In conclusion, EPHA2 promotes the proliferation of Cal-27 cells, inhibits cell apoptosis, and promotes cell migration and invasion by enhancing the AKT/mTOR signaling pathway. Our findings offer a new idea of individualized treatment with AKT/mTOR inhibitors with EPHA2 overexpression for OSCC patients.

## Figures and Tables

**Table 1:** Antibody information.

Antibody	Species	Brand	Cat. no	Dilution
EPHA2	Rabbit	Bioswamp, China	PAB35096	1 : 1000
CyclinA	Rabbit	Abcam, UK	AB53699	1 : 1000
CyclinD1	Rabbit	Abcam, UK	AB40754	1 : 2000
Krp1	Rabbit	Bioswamp, China	PAB39436	1 : 1000
WASP-B	Rabbit	Abcam, UK	AB68182	1 : 1000
Lasp1	Rabbit	Bioswamp, China	PAB33726	1 : 1000
AKT	Rabbit	Abcam, UK	AB8805	1 : 1000
p-AKT	Rabbit	Abcam, UK	AB38449	1 : 1000
mTOR	Rabbit	Abcam, UK	AB32028	1 : 2000
p-mTOR	Rabbit	Abcam, UK	AB84400	1 : 1000
4EBP1	Rabbit	Abcam, UK	AB131453	1 : 1000
p-4EBP1	Rabbit	Abcam, UK	AB75767	1 : 1000
S6K	Rabbit	Abcam, UK	AB32529	1 : 5000
p-S6K	Rabbit	Abcam, UK	AB59208	1 : 1000
GAPDH	Rabbit	Bioswamp, China	PAB36264	1 : 10000
Goat anti-rabbit IgG	Goat	Bioswamp, China	SAB43711	1 : 10000

## Data Availability

The datasets used and/or analyzed during the current study are available from the corresponding author on reasonable request.
